# From intervention to impact: modelling the potential mortality impact achievable by different long-lasting, insecticide-treated net delivery strategies

**DOI:** 10.1186/1475-2875-11-327

**Published:** 2012-09-13

**Authors:** Lucy C Okell, Lucy Smith Paintain, Jayne Webster, Kara Hanson, Jo Lines

**Affiliations:** 1 Department of Infectious Disease Epidemiology, MRC Centre for Outbreak Analysis and Modelling, Imperial College London, Norfolk Place, London, W2 1PG, UK; 2 Department of Disease Control, Faculty of Infectious & Tropical Diseases, London School of Hygiene & Tropical Medicine, Keppel Street, London, WC1E 7HT, UK; 3 Department of Global Health and Development, Faculty of Public Health Policy, London School of Hygiene and Tropical Medicine, 15-17 Tavistock Place, London, WC1H 9SH, UK

## Abstract

**Background:**

The current target of universal access to long-lasting, insecticide-treated nets (LLIN) is 80% coverage to reduce malaria deaths by 75% by 2015. So far, campaigns have been the main channel for large-scale delivery of LLINs, however the World Health Organization has recommended that equal priority should be given to delivery via routine antenatal care (ANC) and immunization systems (EPI) to target pregnant women and children from birth. These various channels of LLIN delivery are targeted to children of different ages. Since risk of mortality varies with child age and LLIN effectiveness declines with net age, it was hypothesized that the age at which a child receives a new LLIN, and therefore the delivery channel, is important in optimizing the health impact of a net.

**Methods:**

A simple dynamic mathematical model was developed of delivery and impact of LLINs among children under five years of age and their household members, incorporating data on age-specific malaria death rates, net use by household structure, and net efficacy over time.

**Results:**

The presented analysis finds that supplementing a universal mass campaign with extra ANC delivery would achieve a 1.4 times higher mortality reduction than campaign delivery alone, reflecting that children born in the years between campaigns would otherwise have access to old nets or no nets at an age of high risk. The relative advantage of supplementary ANC delivery is still present though smaller if malaria transmission levels are lower or if there is a strong mass effect achieved by mass campaigns.

**Conclusion:**

These results indicate that LLIN delivery policies must take into account the age of greatest malaria risk. Emphasis should be placed on supporting routine delivery of LLINs to young children as well as campaigns.

## Background

Long-lasting, insecticide-treated nets (LLINs) are one of the most efficacious preventive interventions against malaria morbidity and mortality available [1] and form a cornerstone of the Roll Back Malaria (RBM) Partnership’s scaling-up for impact strategy to reduce malaria-related mortality by 75% from 2000 levels by 2015 [2]. To achieve this level of impact, RBM has set the target of reaching and sustaining 80% universal coverage with LLINs, meaning that 80% of all members of populations at risk of malaria should be sleeping under an ITN. Although this represents a move away from the previous emphasis on targeting pregnant women and children under five years, these vulnerable groups are still a priority for control programmes [2].

The RBM strategic plan recommends that the 80% universal coverage target is achieved using a combination of campaigns and continuous channels such as routine antenatal clinics (ANC) and the expanded programme of immunization (EPI) for LLIN delivery [2], the so-called “catch-up” and “keep-up” approach [3]. According to the 2011 World Malaria Report, 38 African countries have adopted a policy of LLIN distribution through ANC clinics, 29 through EPI clinics, and 36 through mass campaigns (not mutually exclusive figures) [4]. In practice, largely due to infrastructural challenges and equity concerns, significant emphasis has so far been placed on mass campaigns through which hundreds of millions of LLINs have been distributed in sub-Saharan Africa since 2002.

Despite these tremendous efforts, emerging data indicate that use of LLINs is not sustained at high levels over time. For example, in Togo and Sierra Leone, the percentage of children under five sleeping under a net dropped to around 50% a year after mass distribution campaigns, and was only 25-30% 18 months later [5,6]. In other countries such as Rwanda and Kenya, use has remained constant for a longer period after an initial dropout, albeit only half or less of children were using LLINs, which still falls considerably short of the 80% coverage target [5]. Routine delivery can achieve high coverage among vulnerable groups, for example, a study of LLINs distributed free through ANC in one district in Uganda showed 99% retention and use seven months after distribution [7]. Household ownership and use of LLINs by children under five both approximately doubled to around 80% and 60%, respectively, following free distribution to infants at completion of their EPI schedule in Malawi; no significant improvement in either indicator was seen in a comparison district [8].

Nationally representative data on LLIN ownership and utilization used to measure progress towards the RBM targets is collected via standard RBM malaria modules included in Demographic and Health Surveys (DHS), UNICEF Multiple Indicator Cluster Surveys (MICS) and Malaria Indicator Surveys (MIS). However, questions on the source of LLINs being used by household members is a relatively recent addition and very few countries, therefore, have data on the relative contribution of campaigns and specific routine or continuous delivery channels (e g, ANC, EPI, school- or community-based delivery) to LLIN ownership [9]. Thus, although the RBM strategic plan calls for a combination of routine and campaign delivery, there are currently few publicly documented studies or datasets from which strategy and implementation questions can be answered.

Coverage achieved by different delivery systems will influence the potential impact on mortality that LLIN programmes can have, likewise the well-documented discrepancy between ownership and use [10]. Universal campaigns can at least initially achieve high coverage across the population, reducing transmission and potentially achieving high vector mortality, thereby protecting even those in the community not sleeping under nets. However, in addition to coverage, the timing at which LLINs reach children may also have important implications for optimizing mortality impact since risk of mortality and LLIN effectiveness are not constant parameters over time: risk of death from malaria tends to decrease with a child’s age in areas of high transmission, while the mortality burden may peak in older age groups as malaria transmission intensity decreases [11]; and an LLIN gradually loses insecticide and gains holes, making it less effective over time [12]. This study therefore hypothesized that the greatest potential impact on mortality will be achieved by covering children with a new LLIN at the age that they are most vulnerable. This may have implications for the optimal choice of delivery system or the delivery system mix.

It is clear from available evidence that campaigns alone are not currently achieving or sustaining the 80% targets for LLIN use [5,13,14]. Conversely, it is unclear how routine delivery can be optimized to complement campaigns, as recommended by RBM. In the context of the Millennium Development Goals (MDG), there is a need to investigate potential achievements beyond improving universal ownership and use of LLINs towards impact on under-five mortality. To answer these questions in the absence of complete empirical data, a simple mathematical model has been designed that uses the best available data to make predictions about the expected impact of different LLIN distribution strategies. In addition to assessing which distribution strategies could maximize mortality impact, the model also explores the potential mortality impact per LLIN delivered, which is an intermediate step towards cost-effectiveness and value for money. Efficiency is increasingly important as malaria-endemic countries and their major health donors face increasing financing constraints for health programmes. For example after years of increase, global funding for malaria levelled off in 2010 compared to 2009 [15].

## Methods

A similar modelling approach to the Lives Saved Tool (LiST) model was adopted [16], where the protective effect of interventions is applied to annual mortality figures to calculate number of deaths prevented. A model was developed describing ownership and use of LLINs among children under five years old and their household members, with an LLIN being received from either routine delivery systems that focus specifically on children under-five (ANC or EPI) or a mass distribution campaign. The impact of LLINs on under five-year-old, malaria-specific, mortality rate over time is estimated over 15 years starting in 2012. Outcomes of interest were computed in six-month time steps: malaria mortality, net distribution, and changes in net efficacy over time (unlike a full transmission model which would need a much shorter time step). Model input parameters are based on data from a number of African countries representing different malaria transmission profiles on demographics, net use in relation to household size and structure, net efficacy and its decline over time. What follows is an overview of the model parameters and construction; further details of the model and equations are given in Additional file 1.

### Model population demographics

**Figure 1:**
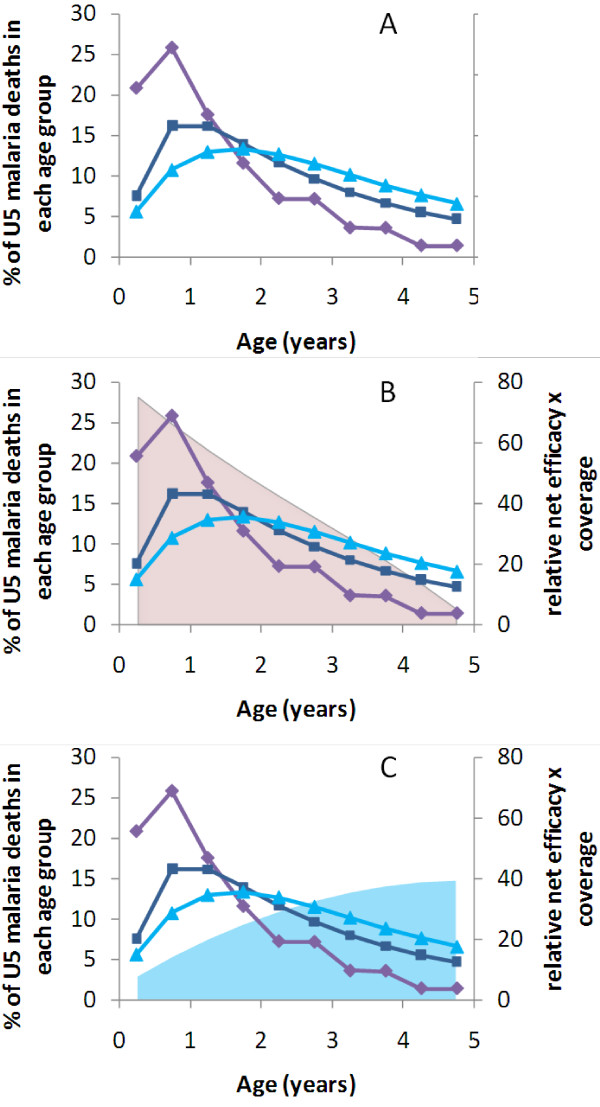
**Age distributions of malaria mortality in three transmission settings, overlaid by the predicted combined effect of net efficacy and coverage attainable by routine *vs* campaign LLIN delivery strategies for each age group.** (**A**) Malaria mortality in a high transmission site [11] (purple diamonds), in a medium transmission site [17] (dark blue squares), and hospital admissions due to malaria in a site with low levels of transmission (light blue triangles) (used as a proxy for distribution of deaths) [17]. (**B**) and (**C**) Mortality patterns together with net efficacy x coverage averaged over five years if (B) LLIN delivered via routine services when child is born (pink area) *vs* (C) if LLIN delivered by campaigns at random ages (light blue area). Calculations assume that once children receive the net they use it for the next three years on average, and that routine service delivery has been in place for more than five years.

The under five-year-old population was modelled in six-month age bands. Three different distributions of age-specific death rates were explored within this group, representing high, medium and low malaria transmission. For the high transmission scenario, age-specific malaria mortality rates averaged across five sites in sub-Saharan Africa were used [11], where malaria mortality peaks in the youngest children and declines as children age towards five years (Figure 1a). These rates were reported per 13 weeks in the first two years of life and per 26 weeks thereafter. For the scenario of medium transmission levels, the malaria-specific death rate distribution identified in a recent review in two medium transmission intensity sites with seasonal transmission was used [17] (Figure 1a). For low transmission settings there is little data on malaria mortality, therefore the age-distribution of malaria-related hospital admissions among under-fives in five sites was used as a proxy in order to test a scenario in which malaria mortality would peak at an older age [17]. For low and medium transmission settings absolute malaria mortality rates in the under five-year-old age group were estimated from Rowe *et al.*[18]. The age-specific death rates and the proportion of children in each age band were assumed to remain constant over time. While immunity to malaria was not explicitly incorporated in the model, the effects of immunity on age patterns of malaria mortality are implicit in the mortality data from endemic areas. It was decided not to model changes in immunity due to the uncertainties in its effects and timescales, although this would be an interesting future question to explore. 

Demographics in older children and adults are not explicitly modelled but coverage and use of LLINs among this group as a whole is tracked in order to model the potential mass effect in the total population acting upon children under five years (see below).

### LLIN impact on mortality

Use of a new LLIN is assumed to reduce malaria mortality by 55% [19]. It is assumed that this relative reduction is constant across age groups in children under five [20] and across transmission intensity settings. A decline in efficacy of the LLIN over time due to decay of the insecticide and incremental damage resulting in holes is modelled. Based on available field data for LLINs [12], efficacy remains high for two to three years then declines more rapidly (Additional file 1: Figure S2). The proportion of deaths averted was assumed to decline proportionately with LLIN efficacy. LLINs were assumed to have a constant impact within each six-month time period, with their efficacy equal to that expected midway through the six-month interval. Loss of nets was modelled as a constant probability of discard over time, with an average LLIN lifespan of three years and a maximum of five years, which is broadly in line with field observations although there are variations between areas [13,21].

### LLIN delivery

Four different delivery strategies for LLIN distribution are included in the model: giving an LLIN (i) to pregnant women at ANC clinic attendance; (ii) to infants attending EPI (routine delivery); or distributing LLINs via mass distribution campaigns, involving (iii) children under five only (targeted campaign); or, (iv) the whole population (universal campaign). Each delivery strategy may be implemented alone or in combination. The assumptions associated with each strategy are described as follows.

### Routine delivery strategies

ANC delivers a LLIN to the mother during pregnancy. It is assumed for simplicity that the LLIN is not used until the child is born [22], i e, a LLIN delivered through ANC is assumed to be zero months old when the child is zero months old. This analysis, therefore, focuses only on the direct protective effect to the newborn infant after birth, not the protective effect on mothers during the pregnancy and associated protection for the unborn infant.

In the model, LLINs delivered via EPI are received by the infant at age six months. With the exception of BCG vaccination, which is scheduled to be administered at birth, EPI visits are scheduled from one to nine months. However, based on a multi-country analysis of actual timing of EPI attendance it is assumed that a high proportion of infants are brought late for their first EPI visit, or do not receive an LLIN until their second EPI visit due to imperfect distribution, giving an average age of six months when an LLIN is received [23]. Once an ANC or EPI LLIN delivery programme has started, coverage is modelled as being constant over time.

### Mass distribution campaigns

A targeted campaign delivers one LLIN to each child under five, while a universal campaign delivers two LLINs to each household. Campaigns are carried out every three years.

### LLIN ownership by individuals and by households

Average LLIN ownership over time was calculated both for children in each six-month age group among the under-fives, and as a total by household, according to the combined coverage of all the delivery strategies described above (see also, Additional file 1). Although children may not personally be given an LLIN via ANC, EPI or a targeted campaign, the model allows for the fact that they may have access to one owned by their household. The analysis was stratified by the number of under-fives in the household. The probability of owning zero, one, or two or more LLINs at each time was calculated for an individual child based on nets obtained from ANC, EPI or targeted campaign delivery channels (Additional file 1: Figure S1). The analysis was stratified by the age of the net. Any child in a household owning more than one net was assumed to be using the newest net [24].

When combining ANC and EPI coverage it was assumed that access to these routine services was not independent, based on data from recent DHS conducted in sub-Saharan Africa (Additional file 1: Table S2) [25,26]. It was assumed that routine and campaign coverage were independent of each other.

Household LLIN ownership (0, 1, 2+) was calculated and stratified by the number of children under five living in the household (0, 1, 2, or 3+) and the age of the newest net. The probability of a child belonging to a household with a given number of under-five children was set according to the household structure recorded in the Tanzania DHS survey in 2005 (Additional file 1: Table S3) [27], since the net use data (Additional file 1: Table S4) was also from Tanzania [28]. Household LLIN ownership depends on the probability of having under-five children who have received LLIN(s) through ANC, EPI and/or targeted campaigns, and the probability of the household having received LLINs through a universal campaign (Additional file 1: Figure S3). Ownership of LLINs from other sources such as schools, community-based strategies and the private sector is not considered.

### Use of LLIN

Data on the reported proportion of individuals sleeping under an LLIN according to the number of children under five present in the household and the number of LLINs owned, was taken from the 2008 household survey of the Tanzania National Voucher Scheme, a detailed dataset available to the authors (Additional file 1: Table S4) [28]. Use of LLINs by under-five children was higher if the household owned multiple LLINs, and if there were fewer other under-five children in the household. Each child was no more likely to use their ‘own’ net than another net owned by the household. It was assumed that the efficacy of the LLIN in reducing mortality was reduced proportionately with use.

### Mass effect

Increased vector mortality resulting from the presence of LLINs in a community can result in an overall reduction in infectious biting, leading to reduced child mortality among individuals not sleeping under an LLIN as well as those directly protected [29,30]. This ‘mass effect’ has been observed in some trials but not in others [1]. Since this parameter has large uncertainty different scenarios were explored in sensitivity analyses.

Number of LLINs owned and use in individuals of all ages was computed as a weighted average, incorporating household structure and its influence on LLIN use (Additional file 1: Tables S3 and S4). This analysis was stratified by the age of the net, assuming that everyone in the household uses the newest LLIN. Although there is some evidence that older children and adults would not use the newest nets [24], this behaviour was not included since the only influence of these older age groups on our outcome of interest, under-five mortality, is via mass effect, which was varied in sensitivity analysis. Mass effect was modelled as a reduction in mortality rate among LLIN non-users achieved by local LLIN coverage among other individuals. The relationship between coverage and mass effect is uncertain and therefore three scenarios were explored (Additional file 1: Figure S4): (i) a mass effect is seen at low LLIN coverage (as suggested by mathematical models e g [29]); (ii) there is almost no mass effect until LLIN coverage is very high; and, (iii) no mass effect. The maximum reduction in mortality among non-users of LLINs achieved by the mass effect was the same as achieved among LLIN users by direct protection.

## Results

### Simple model: individual LLIN ownership by individual LLIN delivery channel

Initially, a simplified version of the model was explored to contrast routine *vs* campaign delivery strategies in the absence of any other LLINs in the household or mass effect. In this calculation, impact is considered over a five-year period in time and it is assumed either that 80% of newborns have received an LLIN from a routine source each year, or that 80% of under-fives received an LLIN from a targeted campaign at the start of the period. To ensure similar numbers of LLINs are distributed in each scenario, it was assumed that there was no repeat campaign delivery during the five-year period, although for the full simulations that follow a three-year campaign cycle was used. Discard of nets is included, and net use is assumed constant at 60% of nets owned [31].

Figure 1 demonstrates how the potential number of preventable deaths essentially depends on when a new LLIN is given in relation to the age of greatest risk of malaria mortality. LLINs delivered to infants through routine services give the greatest efficacy in the youngest age groups, which then declines over time due to loss of efficacy as the net ages and due to discard of nets (Figure 1B). The average efficacy of LLINs among the population of under-five children delivered through a one-off targeted campaign increases with the age of the child (Figure 1C). This is because if the campaign is only carried out every five years (as in this initial simple version of the model), only one in five children will receive an LLIN while still under one year of age. Younger children will then age into the older groups whilst still owning the net. There would be benefits of campaign nets among children over five but the focus of this analysis is only on the under-fives.

**Figure 2:**
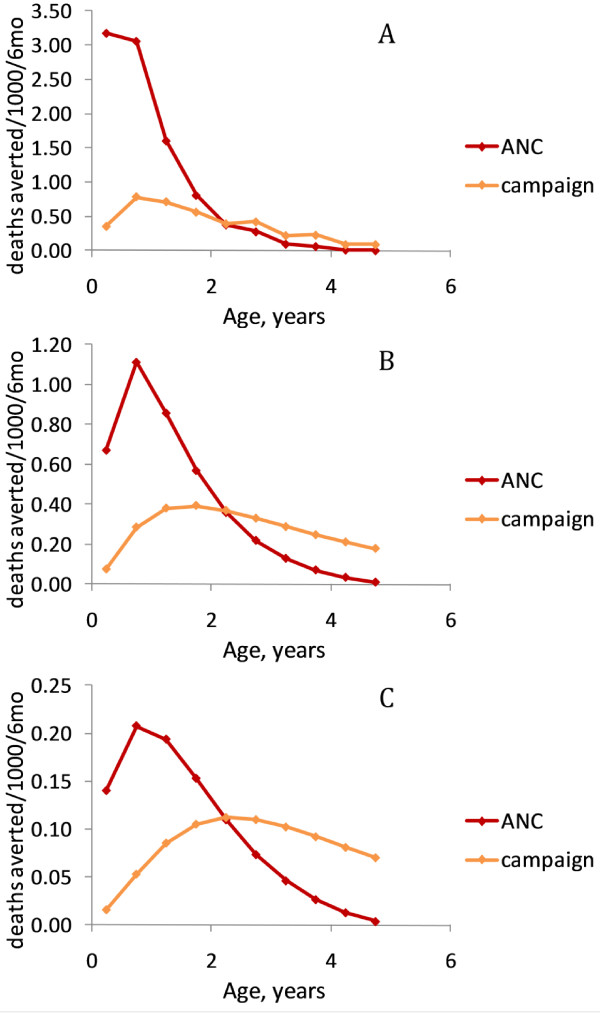
**Mortality impact by age based on a simplified model of individual LLIN ownership with 80% coverage of routine delivery (at birth) or one targeted campaign, average net lifespan of three years and 60% use of LLINs delivered.** Age distributions of malaria mortality are for (**A**) high; (**B**) medium; and (**C**) low transmission intensity (see Figure 1). Ratio of total deaths averted by routine *vs* targeted campaign LLIN delivery is shown on each Figure. Total deaths averted per 1,000 children per six months are: high transmission scenario: routine = 8.50, campaign = 3.85; medium transmission scenario: routine = 3.61, campaign = 2.75; low transmission scenario: routine = 0.86, campaign = 0.83. All numbers are adjusted for number of LLINs delivered.

Routine LLIN delivery at birth predicts more deaths averted among under-fives than a campaign LLIN delivery in high and medium transmission settings (Figure 2A and 2B), while in a low transmission scenario both delivery methods are estimated to have a very similar effect (Figure 2C). This is because for high and medium levels of malaria transmission intensity, a greater proportion of under-five deaths coincide with the period of greatest average LLIN coverage and efficacy for an LLIN delivered at birth (Figure 2). The ratio of under-five malaria deaths averted per LLIN delivered through routine delivery *vs* campaign delivery decreases with transmission intensity (from 2.21 at high transmission intensity to 1.31 and 1.04 at medium and low transmission intensities, respectively) reflecting the shift in risk of malaria mortality to older age groups. However, sharing of LLINs among members of the household and mass effect of the intervention may affect these comparisons of routine *vs* campaign delivery strategies, and these factors are therefore incorporated in the analysis that follows.

### Predicted mortality impact of individual LLIN delivery channels incorporating household structure

**Figure 3:**
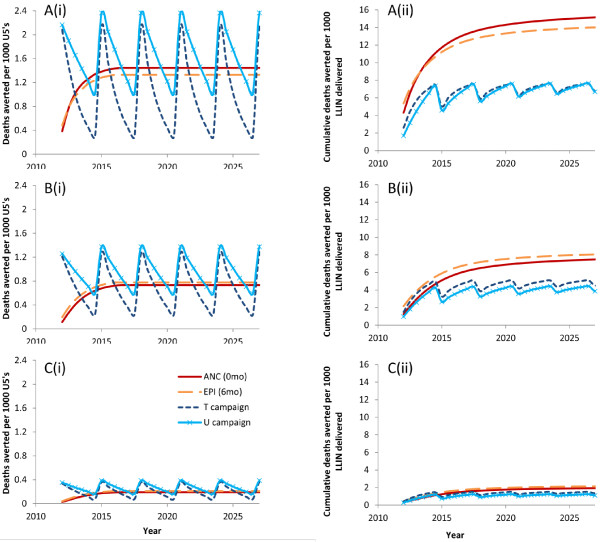
**(i) Deaths averted per six months per 1,000 children under five years; and (ii) cumulative deaths averted per 1,000 LLINs delivered, according to individual delivery channel at (A) high; (B) medium; and (C) low transmission intensity.** New LLIN delivered through ANC to child at age zero months; through EPI to child aged six months; T = targeted campaign, delivering one LLIN per under-five child every three years; U = universal campaign, delivering two LLINs per household every three years; personal protection only i e, no mass effect; 80% ownership for each delivery channel.

The effects of household structure on ownership and use of LLINs by under fives in the household were incorporated to generate the full model. The number of malaria deaths averted per 1,000 children under five every six months and the cumulative under-five deaths averted per 1,000 LLINs delivered were calculated for each delivery scenario. All children were included in these calculations whether they received an LLIN or not. In the base case, the universal target of 80% coverage (taken here to be ownership of 1+ LLINs) was assumed for each delivery channel and any potential mass effect on the vector population was ignored (Figure 3). For the campaign delivery channels, the mortality impact measures are smoothed to provide the mean across the three-year peak and trough campaign cycle.

In all malaria transmission settings, the pattern of malaria deaths averted for routine delivery channels shows a gradual increase initially as infants are covered with an LLIN and age into older age groups, then reaches equilibrium once the first infants to receive the intervention are five years old. The level of this equilibrium and the relative impact of LLINs delivered via ANC compared to EPI differ by transmission intensity, reflecting the different age patterns of malaria mortality. For example, in the high transmission setting, 1.47 deaths per 1,000 children could be averted per six months by LLINs delivered through ANC and used from birth, compared to 1.35 by LLINs delivered through EPI at six months of age (Figure 3Ai); this is because in a high transmission setting, it is assumed that EPI delivery would miss a vulnerable period during the first six months of life. For areas of medium or low transmission, the mortality impact is greater for LLINs delivered through EPI than those delivered through ANC (Figures 3Bi and 3Ci), reflecting the greater mortality risk for slightly older children, although this analysis does not include the indirect benefits of protecting pregnant women by ANC delivery.

The pattern for campaign delivery demonstrates three-yearly cycles of peaks in deaths averted immediately following the campaign, followed by troughs as the efficacy of the LLINs declines and more children are born into the cohort without LLINs. Universal campaigns are predicted to have a slightly higher mortality impact than targeted campaigns (Figures 3Ai, 3Bi and 3Ci) due to the higher number of LLINs delivered; however, the impact per net is lower although the difference is not very large (Figures 3Aii, 3Bii and 3Cii). Both universal and targeted campaign delivery channels show a smaller impact per LLIN delivered than the routine channels. In a high transmission setting, the difference is considerable, with about half the number of deaths among under-fives averted per LLIN delivered by campaign compared to those delivered through ANC or EPI (Figure 3Aii). This pattern is true for all transmission settings (Figures 3Bii, 3Cii). However, campaign nets would have a greater effect on mortality among the over fives, which is not quantified here.

### Sensitivity analysis: impact of mass effect

**Figure 4:**
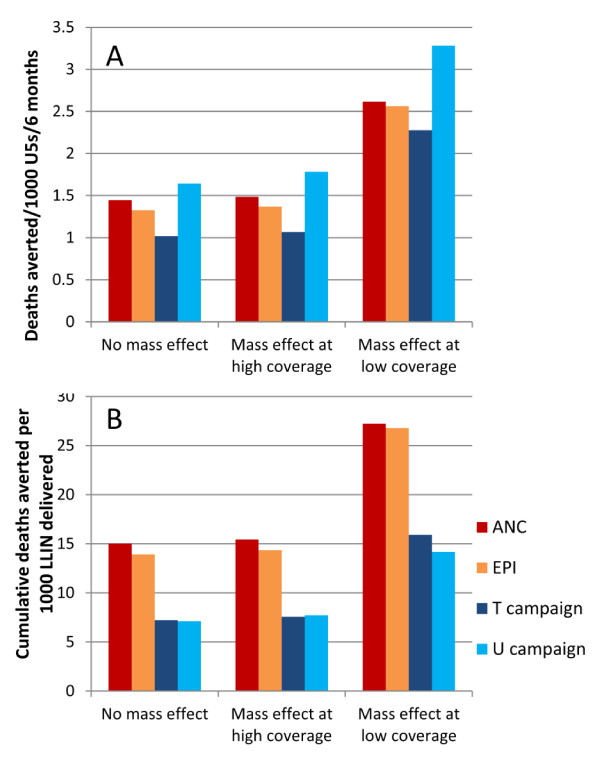
**(A) Deaths averted per six months per 1,000 children under five years; and (B) cumulative deaths averted per 1,000 LLINs delivered in a high transmission setting after 15 years of intervention, accounting for level of mass effect.** For campaign impact, results are averaged over the three-year cycle. T = targeted campaign, U = universal campaign. Each delivery channel delivers LLINs to 80% of people.

The impact of mass effect on predicted number of deaths averted in a high transmission setting was explored 15 years post-intervention when smoothed mortality impact has reached a steady equilibrium. In the scenario where mass effect was present only at high levels of LLIN use, the predicted impact was almost identical to the base case of no mass effect (Figure 4A and 4B). This is because even when coverage in terms of delivering nets is 80%, net discard and lack of net use reduce the potential for mass effect.

In the scenario where a strong mass effect was present at lower levels of LLIN use, the predicted mortality impact was considerably greater when compared to the scenario without any mass effect (Figure 4A). However, the relative impact of routine *vs* campaign delivery strategies is only slightly reduced with increasing mass effect. For example, with no mass effect the predicted mean cumulative number of deaths averted per 1,000 LLINs delivered through ANC is 2.13 times greater than for universal campaigns, while with mass effect at low LLIN coverage, the ratio is 1.92 (Figure 4B). In the model this is because the campaign delivery system results in peaks and troughs in coverage among the population over time, and is able to achieve a mass effect on the vector population during the peak coverage, while routine delivery channels achieve a medium and constant level of coverage, which has less mass effect on the vector population. Similar patterns of predicted mortality impact are found for medium and low transmission settings when the two different patterns of mass effect are explored (results not shown).

### Predicted mortality impact of combinations of routine and campaign LLIN delivery channels

**Figure 5:**
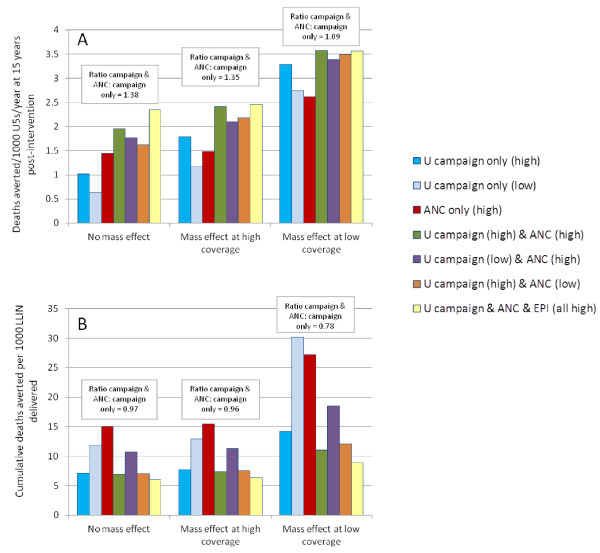
**(A) Deaths averted per 1,000 children under five years; and (B) cumulative deaths averted per 1,000 LLINs delivered after 15 years of intervention, in a high transmission setting for combinations of delivery channel, accounting for level of mass effect.** U = universal campaign; ‘high’ = 80% ownership achieved by delivery channel; ‘low’ = 50% ownership achieved by delivery channel. The text boxes show the ratio of deaths averted (**A**) or the ratio of cumulative deaths averted per 1000 LLIN (**B**) for the campaign alone *vs* the campaign with added ANC, when there is high coverage of both.

Different combinations of routine and campaign delivery channels were explored to predict the potential impact of LLIN delivery strategies under different operational conditions. “High” coverage was set at 80% ownership of one or more nets delivered through the specified delivery channel to reflect the RBM targets for universal coverage; “low” coverage was set at 50% ownership to reflect results reported in the literature from evaluations of campaign and routine delivery programmes. Of particular current interest to policy makers is the magnitude of potential added value if routine continuous LLIN delivery is added to regular universal or targeted campaigns. Three combinations of coverage were therefore explored: high campaign with high routine; low campaign with high routine; high campaign with low routine; these were compared to three-yearly universal campaigns conducted without any routine distribution. In a high transmission intensity setting, adding ANC delivery to a universal campaign results in a 1.4-fold increase in deaths averted compared to campaign delivery alone (Figure 5). The relative impact of adding ANC delivery to a universal campaign *vs* a universal campaign on its own is lower as the assumed level of mass effect increases (Figure 5A). For example, where there is mass effect at low LLIN coverage levels, there is only a 1.1-fold additional effect of adding ANC *vs* campaign only. This is because the campaign has a greater impact on its own when there is strong mass effect.

While the absolute number of deaths averted per 1,000 children is always predicted to increase if multiple delivery channels are modelled (higher numbers of nets are delivered, resulting in higher household LLIN ownership which increases net use) (Figure 5A), impact on deaths averted per 1,000 LLINs delivered decreases due to some overlap in coverage (Figure 5B). Similar patterns were found when transmission intensity was varied.

Higher coverage of routine LLIN delivery channels, whether in combination with low or high universal campaign coverage, maintains a greater number of deaths averted between campaigns (Figure 5B).

Similar patterns were found for the predicted impact of different combinations of delivery channels in areas of medium and low malaria transmission, although total malaria mortality is lower and so the numbers of deaths averted are fewer (results not shown). There were two differences of note. Firstly, in both medium and low transmission scenarios, LLINs delivered through EPI had a greater potential impact than those delivered through ANC, reflecting the greater risk of malaria mortality in older age groups. Secondly, adding ANC delivery to campaign delivery gave a slightly smaller relative gain in low to medium settings although the difference was not great. For example, ANC added to a campaign still increased the number of deaths averted 1.3-fold at low transmission (*vs* 1.4-fold in a higher transmission scenario).

## Discussion

This model supports the hypothesis that the maximum impact of LLINs in terms of reduction in under-five mortality will be achieved if a child receives and uses a new LLIN nearest to the age at which they are at greatest risk of dying from malaria. If delivering LLINs through two different channels, then a combination of one routine delivery channel that gives LLINs to infants together with a targeted or universal campaign is predicted to achieve optimal impact, since new nets reach both younger and older children. This analysis found that delivering nets through additional channels in any combination of ANC, EPI and a targeted or universal campaign always prevented a higher number of deaths (i e, saturation of LLINs in the community was not predicted). If considering ‘efficiency’ where the impact on mortality is presented in relation to the number of LLINs delivered, delivery of a very large number of nets will improve mortality outcomes but reduce efficiency since there will be some overlap in coverage. The balance between effectiveness and efficiency presents a dilemma for control programmes and ministries of health. This model predicts the greatest efficiency for delivery of LLINs through ANC or EPI if only one delivery channel is used; however these routine delivery systems alone do not achieve high enough coverage. Maximum effectiveness is achieved by delivering through as many channels as possible. Perhaps the compromise is LLIN delivery through one routine channel, the choice of which will depend upon context in terms of transmission intensity, together with regular campaigns, which would achieve good levels of effectiveness for a medium level of efficiency.

Nevertheless, the cost per LLIN delivered for each delivery system combination has to be considered. Provider-side financial cost per treated net delivered through routine ANC at district scale ranged from US$7.64 in Kenya [32] to US$8.83 in Uganda [7] and US$8.20 in Burkina Faso [33]. National-level distribution through ANC in Eritrea had an estimated financial cost of US$10.67 per treated net [34]. Provider-side financial cost per treated net delivered through a district-scale targeted campaign ranged from US$3.71 in Tanzania [35] to US$11.53 in Ghana [36] and $10.88 in Zambia [37], where treated net delivery was integrated with immunization campaigns and US$8.30 in Uganda [7] where LLINs were delivered through a stand-alone targeted campaign. Note, all figures are adjusted for inflation and are presented in 2010 US$ [38]. Although difficult to ascertain with confidence due to methodological differences in the studies, the data suggest that LLIN delivery costs are comparable across delivery channels; national-level routine net distributions in Eritrea and Malawi suggest that there may be economies of scale [34,39]. So far there are no published studies of the costs of universal campaigns, which represent a relatively recent shift in policy. Similarly, the authors are not aware of data on costs and effects of combinations of delivery channels

Equity should also be considered, and evidence suggests that the same children may be missed by all interventions [40] due to low socio-economic status or living in remote areas that may be underserved even by mass campaigns. Although socio-economic equity tends to increase with LLIN coverage and will become less of an issue as coverage reaches over 80% [41], sustained coverage of this magnitude is still elusive in most countries. It was considered that ANC and EPI attendance are not independent, with mothers more likely to attend both ANC and EPI or neither [25], however it was assumed that LLIN delivery through routine and campaign channels is independent, which may not be correct, particularly if routine health facilities are utilized for mass campaign distribution. This assumption was made in large part due to a lack of empirical evidence on the level of overlap between children or households reached by routine and campaign LLINs; to overcome this limitation requires data on source of each LLIN in a household, not currently collected in a standard manner by nationally representative surveys. Furthermore correlations between individuals in a household in terms of net receipt or use were not considered.

The results presented here support the RBM-recommended strategy of a combination of routine and campaign LLIN delivery. However, the focus over the last few years has been on campaigns and the process of ‘catch up’ to progress towards the universal coverage goal. Reasonable results have been shown for a number of routine delivery strategies [7,8,28,34], although progress towards ownership and use targets is inevitably slower than that achieved by mass campaign distributions [41], reflecting the complexity of delivering interventions through the infrastructure of a health system [9]. Although many countries have policies for delivery of LLINs through ANC and/or EPI [4], it is not clear that the policies are effectively implemented in all countries. The model predicts that without the contribution of routine LLINs then the mortality impact achievable in the under-five group by campaigns alone is less impressive, particularly in the years between campaigns, and achievement of the mortality MDG becomes less likely. More information is needed on the facilitating factors and barriers to successful LLIN distribution through routine channels. In addition, the question remains of how to reach the children likely to be missed by both routine and campaign channels; any LLIN delivery strategy is likely to need some mechanism of extended outreach for covering hard-to-reach individuals and communities [40,42].

This analysis used a simple model, which has the advantage of greater transparency and fewer assumptions about uncertain factors, but also has limitations. For example, the data used to model household structure and its influence on net use is specific to Tanzania and this may show different patterns in other countries with alternative household structures; it would be of interest to explore the effect of altering these parameters on model predictions in future analyses. The model also does not allow for changing immunity profiles as a result of LLIN introduction, which may shift mortality burden to older age groups. This factor was not included because the timescale over which immunity is lost is highly uncertain, and therefore any model dependent on this would be extremely reliant on which assumptions were made. So far there is no evidence that LLINs do shift mortality to older ages [43-45]. However if such a change did occur, having good coverage of mass campaigns in combination with routine services would be especially important to ensure older as well as younger children access new and efficacious nets. Benefits to members of the population over five years old are not quantified, which may be important with declining malaria transmission levels in many countries. However the results are valid for under five-year-olds, and the decision to focus on this group reflects the importance of health impact for children under five in the early randomized controlled trials that demonstrated the potential of insecticide-treated nets as a highly effective malaria control tool, and the continued MDG focus on reducing child mortality as well as focus of health indicators on the under fives. The focus on under-fives has particular implications for the interpretation of the predicted impact of targeted campaigns, where further benefit was not tracked once the child is older than five years, nor use of the LLIN by others in the household after this time. Furthermore, the focus on under-five mortality only may underestimate the benefit of universal campaigns in comparison to campaigns targeted to the under fives [10]. Ideally the model would be fitted to mortality reductions observed over time in one or more large-scale LLIN delivery programmes. However, a suitable dataset would require detailed data on LLIN distributions, household ownership, usage and mortality over time, which to our knowledge is not available in any existing dataset.

A constant relative risk of malaria mortality among LLIN users *vs* non-users was assumed throughout the under-five cohort, as supported by evidence from an intervention trial [20]. However in a different trial, insecticide-treated nets appeared to have a larger effect among younger children (<3 years) [46]. If this is the case, the results presented in this analysis would underestimate the difference between the impact of ANC and EPI, compared to campaign delivery. This analysis also does not quantify benefits to the mother (or newborn) due to prevention of malaria in pregnancy by the use of an LLIN and, therefore, the benefit of ANC delivery is likely to be underestimated. The use of LLINs during pregnancy has been shown to increase mean birth weight and reduce the risk of miscarriages or stillbirth [47]. Although maternal malaria appears to influence the risk of infant mortality [48], this relationship is complex and the true magnitude of an effect or the potential reduction in infant mortality that may be achievable by LLIN use during pregnancy is unclear [19,49]. Therefore, these factors were not included in the current model in order to avoid the introduction of additional uncertainty and complexity.

One of the arguments for the expansion of LLIN delivery to all age groups rather than only the biologically vulnerable is the potential reduction in transmission and related health burden at the community level (the mass effect), which has been demonstrated by field data [30,50] and supported by mathematical models [29,51]. The inclusion of mass effect in this model goes some way to investigating the influence of universal coverage strategies on predicted health impact. Nevertheless, uncertainties remain around the threshold of LLIN use that is needed to achieve benefits for non-users [29] and the present model predictions were sensitive to the inclusion and pattern of mass effect. The magnitude of impact was larger and the difference in impact between routine and campaign delivery was smaller when mass effect was included. More empirical field data is needed to understand the magnitude of benefits for non-users at different levels of LLIN use.

There is some evidence that the proportional reduction in all-cause under-five mortality achieved by insecticide-treated nets is higher at low transmission intensities [1] but this variation is not well quantified and therefore was not included. While the exact mortality rate ratio affects the estimate of the number of deaths averted, it does not affect comparison of the relative impact of different delivery strategies. Malaria mortality is notoriously difficult to measure, since malaria symptoms resemble other illnesses and malaria may exacerbate other health conditions, causing indirect deaths; therefore, while the parameters in this model are based on detailed reviews [11,17-19], the absolute number of deaths prevented should be interpreted cautiously. Nonetheless, this analysis should provide a robust comparison of different strategies and broadly contrast areas where malaria deaths peak in younger *vs* older children. We assumed homogeneous conditions across the modelled population, for example regarding transmission intensity and use of nets. To capture a more realistic heterogeneous population, for example to model potential impact of campaigns targeted at particular geographical areas, it would be necessary to vary these factors in order to represent sub-populations of interest.

Previous mathematical models have examined the effects of LLINs on malaria transmission at a population level, using a full transmission model framework and focusing on reductions in prevalence of infection and effects on the vector population [51-53]; different modes of delivery and lifespan and efficacy of nets have also been considered [53]. In this analysis a simpler framework is used and focus is specifically on mortality outcomes and the influence of overlap in net efficacy and age-specific mortality patterns within a vulnerable group, the under fives. Future work will incorporate these factors and outcomes within a full transmission model framework, enabling exploration of a range of outcomes in different age groups and the influence of dynamic changes in immunity and mortality impact of LLINs over time as transmission is reduced.

## Conclusion

Mathematical modelling confirms that in order to maximize the number of deaths of children under five that can be averted by LLINs, delivery through routine ANC and/or immunization services is necessary in addition to delivery through campaigns. Regardless of the level of mass effect, average cumulative health impact (mean proportion of deaths averted per 1,000 LLINs delivered) was greater with LLINs delivered to an infant through ANC or EPI than with LLINs delivered through campaigns alone. This reflects that when a new LLIN is delivered to a child at their age of greatest risk of malaria mortality, the period of greatest vulnerability in the child coincides with the period of greatest protective efficacy in the net. Strong emphasis should be placed on supporting routine delivery of LLINs and not focusing resources purely on campaigns. A transition from a campaign focus to one balanced with routine service delivery will require a well-coordinated and funded effort between national programmes and their partners to assure well-functioning infrastructure for routine delivery is in place. More lessons need to be collated and capacity strengthened for effective delivery of LLINs through routine channels.

## Competing interests

The authors declare that they have no competing interests.

## Authors’ contributions

JL, LSP, JW and KH devised the study design and objectives. LO developed the model. LO, LSP, JL, JW and KH contributed to parameterization, analysis and interpretation. LSP and LO wrote the first draft of the manuscript. All authors read, commented on and approved the final manuscript.

## Supplementary Material

Additional file 1 Supplementary information on parameter assumptions and data sources. Click here for file
