# Supplementary material for: From intervention to impact: modelling the potential mortality impact achievable by different long-lasting, insecticide-treated net delivery strategies
Source: Malar J. 2012 Sep 13;11:327. doi: 10.1186/1475-2875-11-327 (PMC3508934; doi:10.1186/1475-2875-11-327)
Supplement: Additional file 1 — Supplementary information on parameter assumptions and data sources. [file 1475-2875-11-327-S1.docx]

**Additional file 1: Supplementary information on parameter assumptions and data sources**

**From intervention to impact: Modelling the potential mortality impact achievable by different LLIN delivery strategies.**

**L.C. Okell, L. Smith Paintain, J. Webster K. Hanson, J. Lines**

**Demographics and impact of LLIN on mortality**

Newly-provided LLIN are estimated to reduce malaria mortality among under-five year olds by 55%, based on cluster-randomized trials [[1](#_ENREF_1)]. We explored scenarios of different distribution of age-specific malaria death rates across under-five age groups which can occur across areas of varying transmission intensity. Firstly we used age-specific malaria mortality rates averaged across five sub-Saharan sites with high transmission: Ifakara and Rufiji in Tanzania, Kourweogo and Oubritenga in Burkina Faso, and Navrongo in Ghana (two further sites in the same publication, Kisumu, Kenya and Manhica, Mozambique, were excluded as they already had ITN in place or lower transmission) [[2](#_ENREF_2)]. Thorough demographic surveillance systems were in place in all sites and malaria deaths were assessed by verbal autopsy. In these high transmission sites, mortality peaks in the youngest children and declines as children age towards 5 years (Figure 1, main text). Data were used at 6-monthly or yearly resolution, as available. In a second scenario, we used the malaria-specific death rate distribution estimated in a recent review in medium transmission intensity sites with seasonal transmission (Figure 1, main text). [[3](#_ENREF_3)]. For our third scenario of low transmission intensity, we used the age-distribution of malaria-related hospital admissions as a proxy for malaria mortality, in order to test a scenario in which malaria mortality would peak at older ages [[3](#_ENREF_3)], since there is very little mortality data in low transmission settings. The medium and low transmission setting data showed the relative distribution of deaths across the under five age groups but lacked denominator information so did not provide a measure of incidence. We therefore used an estimate of the absolute malaria mortality rate from a second study for low transmission sites [[4](#_ENREF_4)]: an average of 2.3 malaria deaths per 1000 person-years among under five year olds in areas where the prevalence of malaria is under 25% (the same cut off as in the first review by Carneiro *et al* [[3](#_ENREF_3)]). We assumed the malaria mortality rate in medium transmission areas to be 8.2 per 1000, the midpoint between the low and high transmission areas.

We assume that the relative reduction in the malaria mortality rate achieved by LLIN is constant across age groups in children under five as suggested by data [[5](#_ENREF_5)]. The proportion of the U5 population in each age group, $c_{a}$, was determined by the all-cause death rate in the preceding age group as measured in [[2](#_ENREF_2)]:

$c_{a}=c_{a-1}e^{-{\tau\mu}_{a-1}}$

where *a* is the index of each age group (1-10), *c* is the proportion of under-five year olds in each age group, $c_{1}$ is the proportion in the age band from 0-0.5 years, $\sum_{a} c_{a}=1$, $\tau$ is the width of the age band in the model (0.5 years) and $\mu_{a}$ is the age-specific annual all-cause mortality rate. All parameters are defined in Table S1. We set the population age structure to be the same in all transmission settings.

The number of deaths averted per 1000 per year among the under-five age group D when LLIN are new and ownership and use are both 100% is therefore given by:

$$D=1000\sum_{a} \tau c_{a}\mu_{a}(1-\psi)$$

where $\psi$ is the malaria mortality rate ratio among LLIN users compared to non-users.

**Table S1: Summary of model parameters, values and variables**

| Parameter | Description | Value in literature | Value(s) used in model |
| --- | --- | --- | --- |
| $\mu$ | Annual malaria mortality rate per 1000 children under 5 years old  Low transmission  Medium transmission  High transmission | 2.3 [[4](#_ENREF_4)]  -  14.1 [[2](#_ENREF_2)] | 2.3  8.2  14.1 |
| $\mu_{a}$ | Annual malaria mortality rate in age group *a* per 1000 | See Figure 1  [2-3] | See Figure 1 |
| $c_{a}$ | Proportion of the U5 population in age group *a* | - | Calculated from death rates $\mu_{a}$ |
| $\psi$ | Malaria mortality rate ratio of LLIN user: non-user when the LLIN is new | [[1](#_ENREF_1)] | 0.45 |
| $\tau$ | Width of age bands, years | - | 0.5 |
| w_A_ | Average age of child in years when mothers start using LLIN delivered by ANC | [[6](#_ENREF_6)] | 0 |
| w*_E_* | Average age of child in years when attending EPI | [[7](#_ENREF_7)] | 0.5 |
| $\rho_{AE}$ | Correlation coefficient of ANC and EPI combined coverage | DHS surveys (Table S2) | 0.51 |
| $\sigma_{s}$ | Relative efficacy of an LLIN of age *s* years, compared to a new LLIN | [[8](#_ENREF_8)] | See text |
| $\gamma$ | Probability of using an LLIN, stratified by household structure and number of LLIN owned | TNVS data | (Table S4) |
| *m* | Parameter controlling rate of decay of LLIN efficacy | - | 4 |
| *k* | Probability of discarding LLIN during a 6–month period | - | 0.114 |
| *q* | Proportion of malaria deaths averted among non-users of LLIN due to mass effect | [[9](#_ENREF_9)] | 0 to 0.55 dependent on *f* |
| *f* | Maximum reduction in mortality among non-users of LLIN due to mass effect as a proportion of the reduction among LLIN users (1-$\psi$) | - | 0, 1 |
| $\beta$ | Parameter controlling the rate of increase of mass effect as LLIN coverage increases | - | 4 |
| Model variable |  |  |  |
| *t* | Time in years |  |  |
| *D* | Deaths averted in all under fives per 1000 per year | - | - |
| $\chi_{A},\chi_{E},\chi_{T},\chi_{U}$ | Coverage of ANC, EPI, targeted campaigns or universal distribution campaigns, respectively (proportion who receive a net) | - | - |
| *a* | Age group (indexed 1-10, representing six month age bands from age 0-5 years) | - | - |
| *s* | Age of LLIN, years | - | - |
| $y_{T},y_{U}$ | Year in which the most recent targeted campaign or universal campaign respectively, was carried out | - | - |
| $p_{A}\left( a \right)$, $p_{E}\left( a \right), p_{T}\left( a \right)$ | Probability of a child of age *a* owning an LLIN which was obtained from ANC, EPI or targeted campaigns, respectively | - | - |
| $p_{U}$ | Probability of a household owning LLIN from a universal campaign | - | - |

**Calculating LLIN ownership of LLIN targeted to children under five years**

We calculated the probability of a single under-five (U5) child having received 0, 1 or 2+ LLIN due to the individual strategies which deliver one LLIN per U5 child (ANC, EPI and targeted campaigns, denoted by subscripts A, E and T, respectively) as follows. Coverage is defined as receipt of an LLIN, therefore incorporating attendance, offer and acceptance.

Firstly we considered ownership of LLIN received from each delivery route separately. The probability of an U5 child in age group *a* owning an LLIN which had been delivered by ANC, $p_{A}\left( a \right)$, when coverage of ANC is constant over time is:

$p_{A}\left( a \right)=0$ where $a<w_{A}$

$p_{A}\left( a \right)=\chi_{A}$ where $a=w_{A}$

$p_{A}\left( a \right)=p_{A}\left( a-1 \right)(1-k)$ where $a>w_{A}$

where $\chi_{A}$ is the coverage of ANC, w*_A_* is the age group of the child when mothers attend ANC, *k* is the probability of discarding the LLIN in each six month time step and $p_{A}\left( a-1 \right)$ denotes the probability of LLIN ownership in the previous six month age band. The probability of an U5 child in age group *a* owning an LLIN due to EPI is calculated exactly as for ANC, with the *A* subscript replaced E to denote the EPI-specific coverage and age of delivery.

The probability of an U5 child owning an LLIN at time *t* obtained from each single, targeted campaign is:

$p_{T}\left( t \right)=0$ where $t-y_{T}>5$

$p_{T}\left( t \right)=\chi_{T}(t)$ where $t=y_{T}$

$p_{T}\left( t \right)=p_{T}\left( t-0.5 \right)(1-k)$ where ${t>y}_{T}$ and $t-y_{T}>5$

where $\chi_{T}(t)$ is the coverage of the targeted campaign at time *t* and $y_{T}$ is the year in which the last campaign was carried out.

We assumed that the average useful LLIN lifespan was 3 years, and that in all cases the net was discarded a maximum of 5 years after delivery, which is broadly in line with field observations although there are variations between areas. The probability of LLIN being thrown away was set at 0.114 per 6-month time step to achieve the average lifespan of 3 years. Benefits of LLIN to children who age out of the under-five age group while still possessing an LLIN are not quantified in this analysis.

Secondly we calculated the age- and time-specific probabilities of a U5 child having received and still retaining 0, 1, or 2+ LLIN through combined effects of ANC, EPI and targeted campaigns. A simplified probability tree of this process is shown in Figure S1. The probability of a child in each age band and in each year having received the number of LLIN indicated through a given combination of delivery channels is obtained simply by multiplication across the appropriate branches of the probability tree. The analysis was also stratified by the age of the net (see below). We did not track numbers of LLIN owned greater than 2 since LLIN use was found to be almost identical among those owning 2 LLIN as those owning 3+ LLIN (Table S4) [10]. We assumed that each child would receive no more than two LLIN via targeted campaigns during their first five years of life, based on the recommended 3-yearly frequency.

When combining ANC and EPI coverage we included the fact that general level of access to healthcare of each individual would lead to a greater overlap in coverage than expected if coverage of each intervention were independent of the other. Based on data from recent Demographic and Health surveys conducted in sub-Saharan Africa (Table S2) the median correlation between coverage between the two interventions is 0.51. A correlation coefficient of zero would represent random coverage of each intervention irrespective of the other, for example if ANC coverage was 0.8 and EPI coverage was 0.5, the probability of an individual attending both interventions would be 0.8 x 0.5 = 0.4. A correlation coefficient of 1 would indicate that the same people received both interventions, so in this example all those individuals receiving EPI would also receive ANC (a proportion of 0.5 of the population). The probability of receiving both interventions $p_{AE}$, given this correlation, was calculated as:

$$p_{AE}=\Phi_{2}\left(\Phi^{-1}(p_{A}), \Phi^{-1}(p_{E}), \rho_{AE}\right)$$

where $\Phi_{2}\left(x, y, \rho_{AE}\right)$ is the probability that $X\leq x$ and $Y\leq y$when $X$ and $Y$ have a bivariate normal distribution, with zero means, standard deviations of 1 and correlation $\rho_{AE}$[11].

**Table S2:** Summary of Demographic and Health Survey (DHS) data on ANC and EPI attendance and overlap in their coverage. Combinations of ANC and EPI attendance relate to the most recent pregnancy and youngest child, respectively; ANC attendance is defined as woman attended ANC at least once in the most recent pregnancy; EPI coverage is defined as child aged 12-23 months received DPT1, as a proxy for attending EPI at least once [[12](#_ENREF_12)].

| **Country & year of survey** | **% women attending ANC at least once** | **Median timing of first ANC visit (months)** | **% children aged 12-23 months receiving DPT1** | **% attend both ANC & EPI** | Correlation coefficient between ANC & EPI attendance $\rho_{AE}$ |
| --- | --- | --- | --- | --- | --- |
| Benin, 2006 | 89.0 | 4 | 83.9 | 79.3 | 0.68 |
| Cameroon, 2004 | 83.8 | 4 | 82.5 | 73.4 | 0.53 |
| Chad, 2004 | 45.3 | 4 | 44.4 | 32.7 | 0.72 |
| Congo, 2005 | 88.4 | 3 | 86 | 79.8 | 0.60 |
| Ethiopia, 2005 | 28.5 | 5 | 57.4 | 22.6 | 0.47 |
| Guinea, 2005 | 84.9 | 4 | 76.9 | 73.3 | 0.80 |
| Lesotho, 2004 | 91.7 | 4 | 93.9 | 86.5 | 0.18 |
| Liberia, 2007 | 97.4 | 3 | 75.2 | 74.1 | 0.37 |
| Madagascar, 2004 | 82.8 | 4 | 70.5 | 64.7 | 0.61 |
| Malawi, 2004 | 95.6 | 5 | 94.5 | 91.1 | 0.43 |
| Mali, 2006 | 75.5 | 4 | 82.5 | 66.5 | 0.45 |
| Namibia, 2007 | 95.8 | 4 | 94.4 | 91.1 | 0.40 |
| Niger, 2006 | 47.4 | 4 | 58 | 38.7 | 0.67 |
| Rwanda, 2005 | 95.1 | 6 | 96.4 | 92.8 | 0.62 |
| Senegal, 2005 | 93.8 | 3 | 93.4 | 88.9 | 0.49 |
| Zimbabwe, 2006 | 96.3 | 5 | 76.4 | 74.7 | 0.37 |
| **Median** | **88.7** | **4.0** | **82.5** | **74.4** | **0.51** |

**Figure S1:** Individual child LLIN ownership: probability tree of an under-five child receiving 0, 1 or ≥2 LLIN via different delivery routes over the first five years of a child’s life. Parameters are as defined in the text above. The number of LLIN owned depending on coverage of ANC, EPI and targeted campaigns are shown after each branch (interventions not restricted to happen in this order). It is assumed that a maximum of 2 targeted campaigns would be carried out during the first five years of a child’s life. The analysis was also stratified by the age of LLIN and it was assumed that LLIN were discarded with a constant probability and none were used longer than 5 years (not shown).

**Efficacy of nets**

All analyses on net ownership were stratified by the age of the net, *s* (equal to the time since distribution). The decline in efficacy of the LLIN due to a combination of insecticide efficacy and number of holes in the net was incorporated [[8](#_ENREF_8)]. The protective efficacy of a new net was assumed to have an optimal value $(1-\psi)$ equal to that measured in randomized controlled trials [[1](#_ENREF_1)]. We calculated relative efficacy $\sigma$ over time using a function which resulted in a slow decay initially followed by a more rapid decay, resulting in a relative efficacy of 1 when the net is new and an efficacy close to zero after 5 years, to match observed data. The relative efficacy of a net of age *s* years is given by

$$\sigma_{s}=1-e^{-m}(e^{0.2ms}-1)$$

where *m* is a parameter controlling the rate of decay in efficacy.

The number of deaths averted was assumed to decline proportionately with LLIN efficacy.

**Figure S2:** LLIN efficacy over time relative to a new LLIN.

**Household LLIN ownership**

Although individual U5 children may not receive LLIN through the channels above, we allowed that they may have access to LLIN in their household received either through universal LLIN campaigns or through living with other U5 children who have received LLIN through ANC, EPI or targeted campaigns. For universal LLIN distribution campaigns, we model a scenario where 2 LLIN are delivered per household. Therefore the probability of a household owning 2 LLIN at time *t* obtained from each single, universal campaign is:

$p_{U}\left( t \right)=0$ where $t-y_{U}>5$

$p_{U}\left( t \right)=\chi_{U}(t)$ where $t=y_{U}$

$p_{U}\left( t \right)=p_{U}\left( t-0.5 \right)(1-k)$ where ${t>y}_{U}$ and $t-y_{U}>5$

where $\chi_{U}(t)$ is the coverage of the universal campaign at time *t* and $y_{U}$ is the year in which the last universal campaign was carried out. We assume that both nets distributed to each household are used concurrently and are equally likely to be discarded at any time.

Total LLIN ownership by a household depends in part on the number of U5 children. We stratify households by 0, 1, 2 or 3+ U5 children (Table S3). For households with 3+ U5’s we assume an average of 3 children based on Demographic & Health Survey data for Tanzania in 2004-5 [[13](#_ENREF_13)], where only 9% of households in the 3+ U5’s category have more than 3 U5 children. These assumptions would not be applicable in areas with a different household structure.

We calculated the probabilities of households having received and retaining 0, 1 or 2+ LLIN through universal campaigns and through delivery channels targeting U5 children living in the household. Figure S3 shows a simplified probability tree from the perspective of a child under five, indicating the probability of living in a household which has received 0, 1 or 2+ LLIN which he/she can access in addition to any of their own. Combining results from probability trees in Figures S1 and S3 we obtain the probability of a child being in a household with 0, 1 or 2+ LLIN due to all delivery channels. Individual household structures and the ages of all the children within them are not modeled explicitly, however the probability that the child is in a household containing a given number of other U5 children is known (see below). Household ownership of LLIN is then calculated based on the average U5 child. As before the analysis is stratified by the age of the LLIN, and we assume that LLIN are discarded after 5 years.

**Use of LLIN by U5**

The probability of using an LLIN if one or more is owned, $\gamma$, was based on data from Tanzania stratified by household structure (0, 1, 2 or 3+ U5 children in the household) and by LLIN ownership at the household level (0, 1 or 2+ LLIN owned) (Table S4) [10]. We used the household structure recorded in the Tanzanian Demographic & Health Survey 2004-2005 to obtain numbers of U5 children living in households of given sizes (Table S3). The probability of a child belonging to a household of a given structure owning a given number of total LLIN was calculated by combining the child’s LLIN with the household LLIN. We assumed that the newest LLIN in the household was always used [[14](#_ENREF_14)]. Each child was no more likely to use their own net compared to any other net owned by the household.

**Table S3**: Distribution of households, children under five and the whole population living in each type of household structure (categorised according to number of children aged under-five (U5) per household) (Source: Tanzania DHS survey 2004-5 [[13](#_ENREF_13)])

| No. children under five (U5) in household (HH) | Proportion living with each HH structure | | |
| --- | --- | --- | --- |
|  | Households | Children under five | Whole population |
| HH with zero U5 | 40.9 | 0 | 27.4 |
| HH with 1 U5 | 30.1 | 30.1 | 30.7 |
| HH with 2 U5 | 20.6 | 41.2 | 26.1 |
| HH with 3 or more U5 | 8.4 | 28.8 | 15.8 |

**Table S4:** Proportion of children under five (U5) and whole population sleeping under an LLIN according to household structure and household LLIN ownership (Source: TNVS, Kara Hanson, personal communication).

| Household (HH) LLIN ownership | N (people) | % of people sleeping under an LLIN | | | | |
| --- | --- | --- | --- | --- | --- | --- |
|  |  | All HH | HH with zero U5 | HH with 1 U5 | HH with 2 U5 | HH with 3 or more U5 |
| Children under five years old | | | | | | |
| HH with 1 net | 1804 | 48.3 | 0 | 58.7 | 43.1 | 39.3 |
| HH with 2 nets | 1376 | 72.9 | 0 | 77.3 | 74.5 | 62.6 |
| HH with >=3 nets | 937 | 71.7 | 0 | 77.4 | 74.3 | 61.2 |
| Whole population | | | | | | |
| HH with 1 net | 7777 | 38.1 | 39.4 | 44.4 | 31.0 | 33.7 |
| HH with 2 nets | 6162 | 64.6 | 68.2 | 67.6 | 64.1 | 47.3 |
| HH with >=3 nets | 5674 | 73.3 | 83.0 | 74.4 | 66.2 | 48.8 |

**Figure S3:** Household LLIN ownership: probability tree of a child having access to 0, 1 or ≥2 LLIN within their household in addition to any received themselves. Up to 2 universal campaigns in which 2 LLIN are delivered per household may be carried out during the first 5 years of a child’s life. If one or two other under-five children live in the household and have received 0,1 or 2 LLIN via ANC, EPI or targeted campaigns then the probabilities are also multiplied across one or both of the last two sections of the probability tree. The probabilities of these other under-five children owning 0, 1 or ≥2 LLIN are obtained from Figure S1 and are a weighted average over the under-five age group. The analysis was also stratified by the age of LLIN and it was assumed that LLIN were no longer used after 5 years (not shown).

**Mass effect**

Increased vector mortality resulting from the presence of LLIN in a community can result in an overall reduction in infectious biting, leading to reduced mortality among individuals not sleeping under LLIN as well as those directly protected [9, 15]. This ‘mass effect’ has been observed in some trials but was absent in others [[16](#_ENREF_16)] and therefore we explored different scenarios.

The relative reduction in malaria mortality achieved in non users of LLIN due to mass effect was denoted *q*. The maximum reduction in mortality among non-users of LLIN achieved by the mass effect was described as a proportion *f* of the reduction estimated to occur among LLIN users $1-\psi$. Coverage and use of LLIN in individuals of all ages was simulated based on data on household structure and its influence on LLIN use (Tables S3 & S4). The proportion of households owning 0, 1 or 2+ LLIN was assessed using the probability trees in Figures S1 and S3, calculating coverage according to the number of universal campaigns targeted at a household level and the number of U5 children within the household who may have received targeted nets. This analysis was stratified by the age of the newest net, assuming that everyone in the household uses the newest LLIN. We assumed that only LLIN which are in use would contribute towards a mass effect, which is conservative since unused LLIN which are hung may still cause some vector mortality.

Since the relationship between coverage and mass effect is uncertain, three scenarios were explored (Figure S4):

1. A mass effect is seen at low LLIN coverage (as suggested by mathematical models e.g. [[9](#_ENREF_9)])

$q=\left( 1-\psi\right)fe^{-\beta\chi\gamma}+\left[ 1-\left( 1-\psi\right)f \right]$

where $\beta$ controls the rate of increase of mass effect as coverage increases.

1. There is almost no mass effect until coverage is very high

$$q=\left( 1-\psi\right)f\left[ 1-e^{-\beta}(e^{\left( 1-\chi\gamma\right)}-1) \right]+\left[ 1-\left( 1-\psi\right)f \right]$$

2. No mass effect.

We assume that an indirect mass effect contributed a negligible amount to the overall 55% reduction in under-five year old mortality measured in field trials. In these trials most children were users of LLIN and there is no clear evidence for additional protection due to mass effect for a child who is already individually protected by an LLIN [[17](#_ENREF_17)].

**Figure S4:** Mass effect: different assumptions about the relationship between local LLIN coverage and reduction in mortality among children under five years old who do not use LLIN. Mortality is shown among LLIN non-users and users relative to a scenario with no LLIN. The maximum effect among non-users is assumed to be the same as or less than the 55% reduction in malaria deaths among LLIN users.

**References**

1. Eisele TP, Larsen D, Steketee RW: **Protective efficacy of interventions for preventing malaria mortality in children in *Plasmodium falciparum* endemic areas.** *Int J Epidemiol* 2010, **39 Suppl 1:**i88-101.

2. Abdullah S, Adazu K, Masanja H, Diallo D, Hodgson A, Ilboudo-Sanogo E, Nhacolo A, Owusu-Agyei S, Thompson R, Smith T, Binka FN: **Patterns of age-specific mortality in children in endemic areas of sub-Saharan Africa.** *Am J Trop Med Hyg* 2007, **77:**99-105.

3. Carneiro I, Roca-Feltrer A, Griffin JT, Smith L, Tanner M, Schellenberg JA, Greenwood B, Schellenberg D: **Age-patterns of malaria vary with severity, transmission intensity and seasonality in sub-Saharan Africa: a systematic review and pooled analysis.** *PLoS One* 2010, **5:**e8988.

4. Rowe AK, Steketee RW: **Predictions of the impact of malaria control efforts on all-cause child mortality in sub-Saharan Africa.** *Am J Trop Med Hyg* 2007, **77:**48-55.

5. Habluetzel A, Diallo DA, Esposito F, Lamizana L, Pagnoni F, Lengeler C, Traore C, Cousens SN: **Do insecticide-treated curtains reduce all-cause child mortality in Burkina Faso?** *Trop Med Int Health* 1997, **2:**855-862.

6. Marchant T, Hanson K, Nathan R, Mponda H, Bruce J, Jones C, Sedekia Y, Mshinda H, Schellenberg J: **Timing of delivery of malaria preventive interventions in pregnancy: results from the Tanzania national voucher programme.** *J Epidemiol Community Health* 2011.

7. Clark A, Sanderson C: **Timing of children's vaccinations in 45 low-income and middle-income countries: an analysis of survey data.** *Lancet* 2009, **373:**1543-1549.

8. Kilian A, Byamukama W, Pigeon O, Atieli F, Duchon S, Phan C: **Long-term field performance of a polyester-based long-lasting insecticidal mosquito net in rural Uganda.** *Malar J* 2008, **7:**49.

9. Killeen GF, Smith TA, Ferguson HM, Mshinda H, Abdulla S, Lengeler C, Kachur SP: **Preventing childhood malaria in Africa by protecting adults from mosquitoes with insecticide-treated nets.** *PLoS Med* 2007, **4:**e229.

10. Hanson K, Marchant T, Nathan R, Mponda H, Jones C, Bruce J, Mshinda H, Schellenberg JA: **Household ownership and use of insecticide treated nets among target groups after implementation of a national voucher programme in the United Republic of Tanzania: plausibility study using three annual cross sectional household surveys.** *BMJ* 2009, **339:**b2434.

11. Griffin JT, Hollingsworth TD, Okell LC, Churcher TS, White M, Hinsley W, Bousema T, Drakeley CJ, Ferguson NM, Basanez MG, Ghani AC: **Reducing *Plasmodium falciparum* malaria transmission in Africa: a model-based evaluation of intervention strategies.** *PLoS Med* 2010, **7:**e1000324.

12. **Measure-DHS. Demographic and Health Surveys.** [**http://www.measuredhs.com/**](http://www.measuredhs.com/)

13. National Bureau of Statistics (Tanzania), ORC Macro: **Tanzania Demographic and Health Survey 2004-05.** In *Book Tanzania Demographic and Health Survey 2004-05* (Editor ed.^eds.). City: National Bureau of Statistics and ORC Macro; 2005.

14. Tsuang A, Lines J, Hanson K: **Which family members use the best nets? An analysis of the condition of mosquito nets and their distribution within households in Tanzania.** *Malar J* 2010, **9:**211.

15. Binka FN, Indome F, Smith T: **Impact of spatial distribution of permethrin-impregnated bed nets on child mortality in rural northern Ghana.** *Am J Trop Med Hyg* 1998, **59:**80-85.

16. Lengeler C: **Insecticide-treated bed nets and curtains for preventing malaria.** *Cochrane Database Syst Rev* 2004**:**CD000363.

17. Howard SC, Omumbo J, Nevill C, Some ES, Donnelly CA, Snow RW: **Evidence for a mass community effect of insecticide-treated bednets on the incidence of malaria on the Kenyan coast.** *Trans R Soc Trop Med Hyg* 2000, **94:**357-360.
